# Hotspots and bottlenecks for the enhancement of the environmental sustainability of pig systems, with emphasis on European pig systems

**DOI:** 10.1186/s40813-023-00347-5

**Published:** 2023-11-16

**Authors:** Georgios Pexas, Ilias Kyriazakis

**Affiliations:** 1 School of Water, Energy and Environment, Cranfield University, Cranfield, UK; 2 Institute for Global Food Security, Queen’s University Belfast, Belfast, UK

**Keywords:** Pig production, Environmental impact, Sustainability, Trade-offs, Environmental impact hotspots, Implementation barriers, Climate change

## Abstract

Although pig systems start from a favourable baseline of environmental impact compared to other livestock systems, there is still scope to reduce their emissions and further mitigate associated impacts, especially in relation to nitrogen and phosphorous emissions. Key environmental impact hotspots of pig production systems are activities associated with feed production and manure management, as well as direct emissions (such as methane) from the animals and energy use. A major contributor to the environmental impacts associated with pig feed is the inclusion of soya in pig diets, especially since European pig systems rely heavily on soya imported from areas of the globe where crop production is associated with significant impacts of land use change, deforestation, carbon emissions, and loss of biodiversity. The “finishing” pig production stage contributes most to these environmental impacts, due to the amount of feed consumed, the efficiency with which feed is utilised, and the amount of manure produced during this stage. By definition therefore, any substantial improvements pig system environmental impact would arise from changes in feed production and manure management. In this paper, we consider potential solutions towards system environmental sustainability at these pig system components, as well as the bottlenecks that inhibit their effective implementation at the desired pace and magnitude. Examples include the quest for alternative protein sources to soya, the limits (perceived or real) to the genetic improvement of pigs, and the implementation of alternative manure management strategies, such as production of biogas through anaerobic digestion. The review identifies and discusses areas that future efforts can focus on, to further advance understanding around the potential sustainability benefits of modifications at various pig system components, and key sustainability trade-offs across the environment—economy—society pillars associated with synergistic and antagonistic effects when joint implementation of multiple solutions is considered. In this way, the review opens a discussion to facilitate the development of holistic decision support tools for pig farm management that account for interactions between the “feed * animal * manure” system components and trade-offs between sustainability priorities (e.g., environmental vs economic performance of pig system; welfare improvements vs environmental impacts).

## Pig systems environmental impact and motivation for improvement

**Fig. 1:**
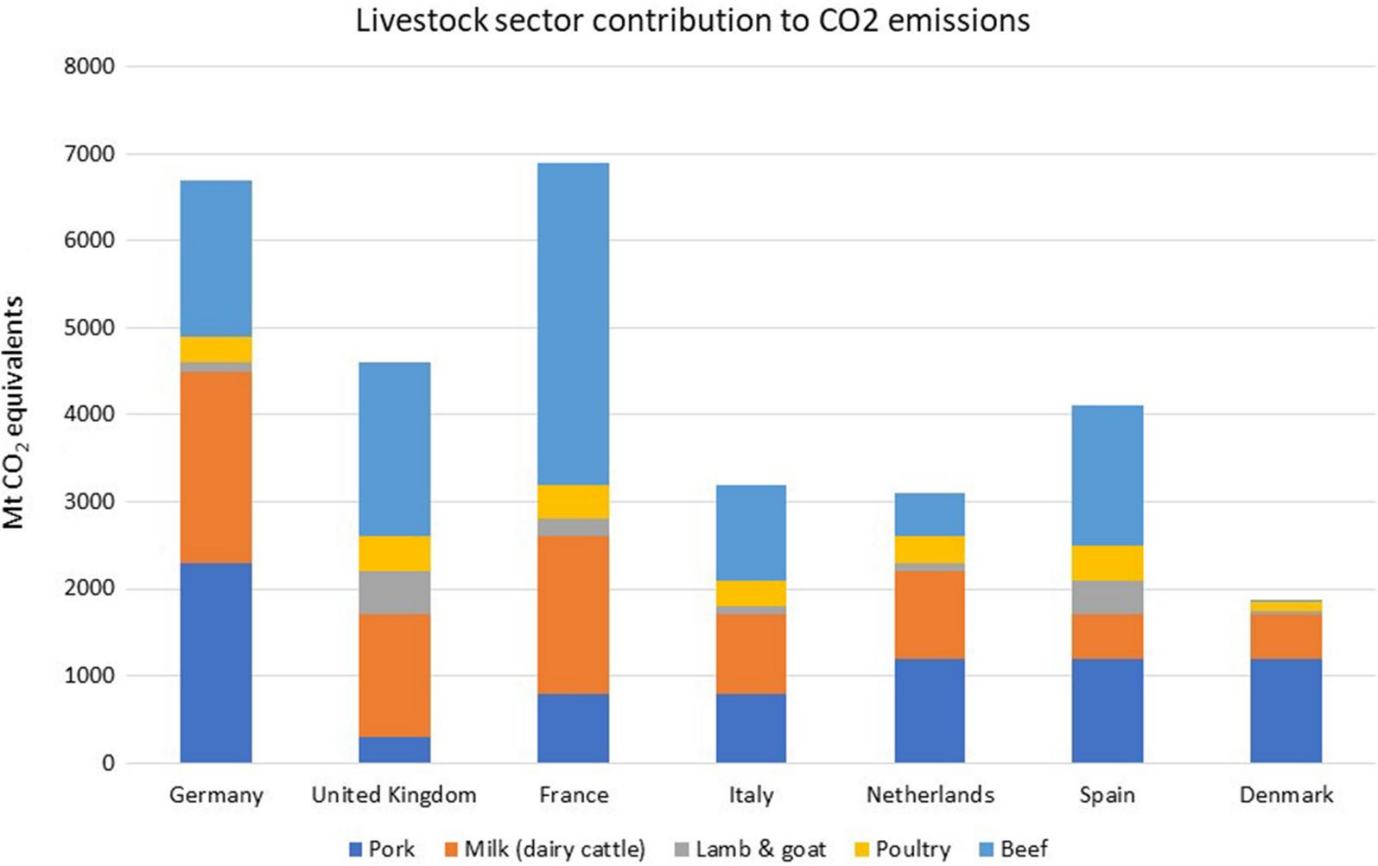
Breakdown of livestock sector contributions to carbon dioxide emissions (Mt CO_2-_ equivalents) across Europe. (adapted from CIEL [14])

With the global population increasing at a steady rate, a consequent increase in livestock meat and milk production and consumption is anticipated [128, 138]. Livestock industries are often criticised for their associated negative environmental impacts and such concerns will only become more relevant considering the potential intensification of the sector [40, 79]. While pig production systems are associated with significantly lower negative environmental impacts than other meat production systems (e.g., beef cattle) (Fig. 1), pork is the most popular meat product globally [14, 41] and therefore not a negligible contributor to environmental impacts arising from livestock production. In their review of the environmental impact arising from livestock systems in OECD countries, De Vries and de Boer [30] found that the production of 1 kg of pork required 8.9–12.1 m^2^ of land, which is significantly lower than beef (27–49 m^2^), but comparable to the production of 1 kg of chicken (8.1–9.9 m^2^). They observed similar trends for other environmental impact categories, including the use of fossil energy (pork 15–45 MJ/kg, chicken 15–29 MJ/kg, and beef 34–52 MJ/kg), global warming potential (pork 3.9–10 kg CO_2_ eq/kg, chicken 3.7–6.9 kg CO_2_ eq/kg, and beef 14–32 kg CO_2_ eq/kg), and acidification (pork 0.002–0.062 kg SO_2_ eq/kg, chicken 0.004–0.022 kg SO_2_eq/kg, and beef 0.008–0.055 kg SO_2_eq/kg) and eutrophication of terrestrial and aquatic ecosystems (pork 0.002–0.02 kg PO_4_ eq/kg, chicken 0.001–0.012 kg PO_4_ eq/kg, and beef 0.004–0.025 kg PO_4_ eq/kg). Therefore, a projected scaling up of pig production with more animals in larger pig farming systems and potentially on or close to currently sensitive areas (e.g., nitrogen and phosphorus vulnerable zones, Natura 2000 ecosystems) is expected to further increase associated environmental burdens and create further issues for environmental and overall sustainability [105].

While carbon dioxide (CO_2_) and methane (CH_4_) emissions, associated with their global warming potential (GWP), are in the core of most research and policy agendas aiming to reduce livestock systems’ environmental footprint, the above wider range of environmental impact categories should be considered in order to secure future sector sustainability [79, 98]. Literature highlights the: i) land use and land use change (LU) including impacts of deforestation and land degradation in sensitive and threatened forested ecosystems (e.g., Latin America), ii) nitrogen and phosphorus related impacts including the potential for acidification (AP) and eutrophication (EP) of terrestrial and aquatic ecosystems, iii) indirect impacts to biodiversity due to habitat fragmentation and direct, iv) depletion of fossil fuel (non-renewable resource use—NRRU), and v) degradation of the quality and depletion of water resources (WRU), as urgent environmental issues to address [2, 30, 79]. Past studies have estimated that pig production contributes approximately 9% of GHG emissions and 25% of ammonia emissions (NH_3_) attributed to livestock production globally; the main GHGs associated with pig production are CH_4_, CO_2_, and nitrous oxide (N_2_O) and are accounted as major contributors to Carbon footprint, GWP, and AP [45, 126]. European benchmarks on the contribution of 1 kg of liveweight (LW) pig meat to acidification and eutrophication of ecosystems report approximately 0.028 kg SO_2_ and 0.025 kg PO_4_ equivalents respectively [98]. Conventional pig systems in Denmark contribute 3.57 kg CO_2_ per kg of LW pig meat [98], in France approximately 2.30 kg CO_2_ per kg of LW pig meat [83], whereas in Midwest US 3.40 kg CO_2_ per kg of LW pig meat [119], and in China close to 4.18 kg CO_2_ per kg of carcass weight (CW) pig meat [69]. These values are considered comparable since key methodological assumptions were consistent across the various Life Cycle Assessment (LCA) studies, namely the cradle-to-farm gate system boundaries, economic allocation of impacts with system expansion to account for potential data and model uncertainties, and impact assessment methods; thereby any exhibited variability is largely attributed to the study functional unit used for calculations (LW vs. CW) and local conditions [145]. Pig meat production is also identified as the third highest water resource using system with 6000 L/kg, after beef (15,400 L/kg) and lamb (10,400 L/kg) [55].

**Fig. 2:**
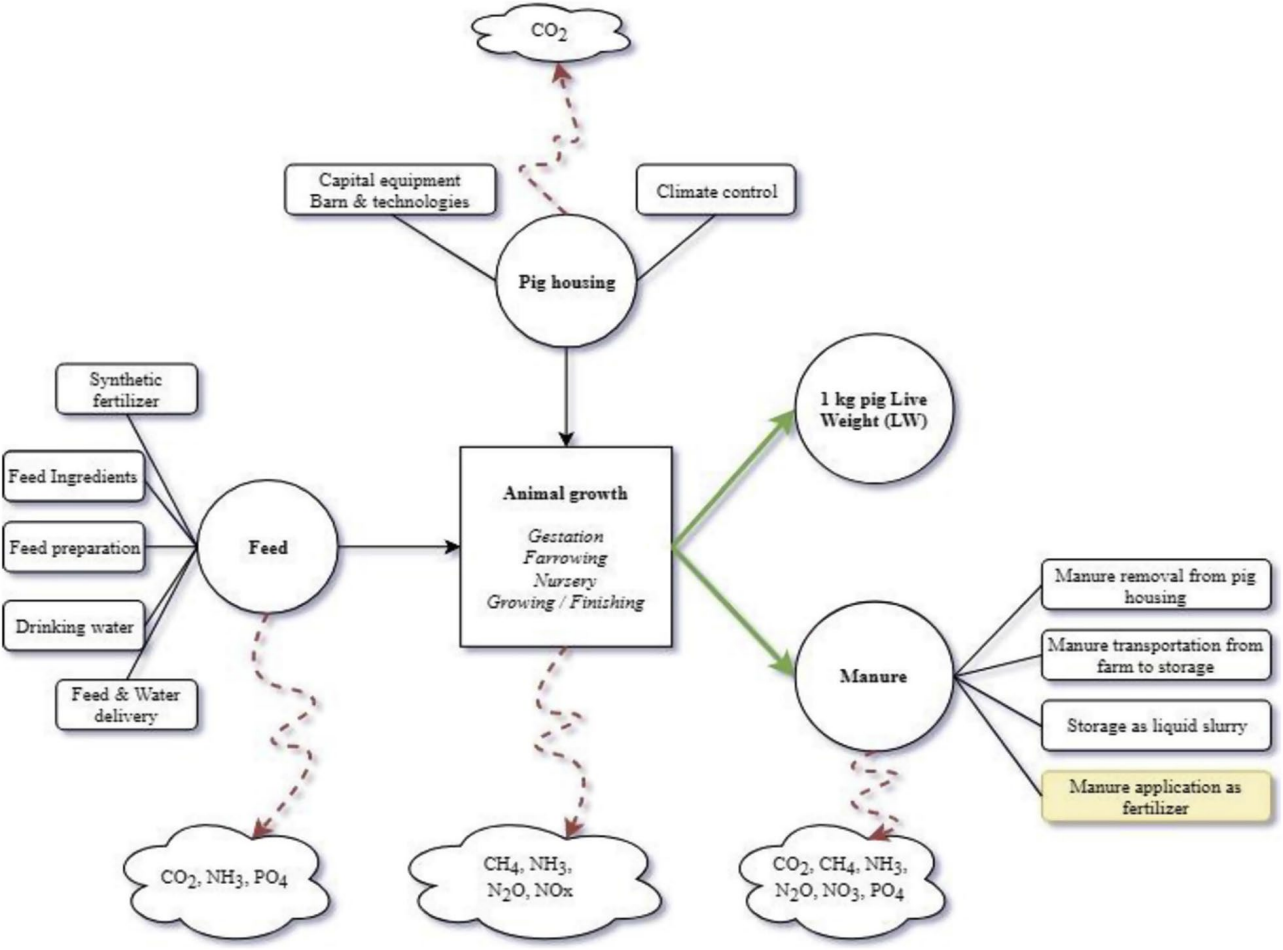
Emission flows through all system components as represented through a Danish conventional pig production system. *Source* [98])

**Fig. 3:**
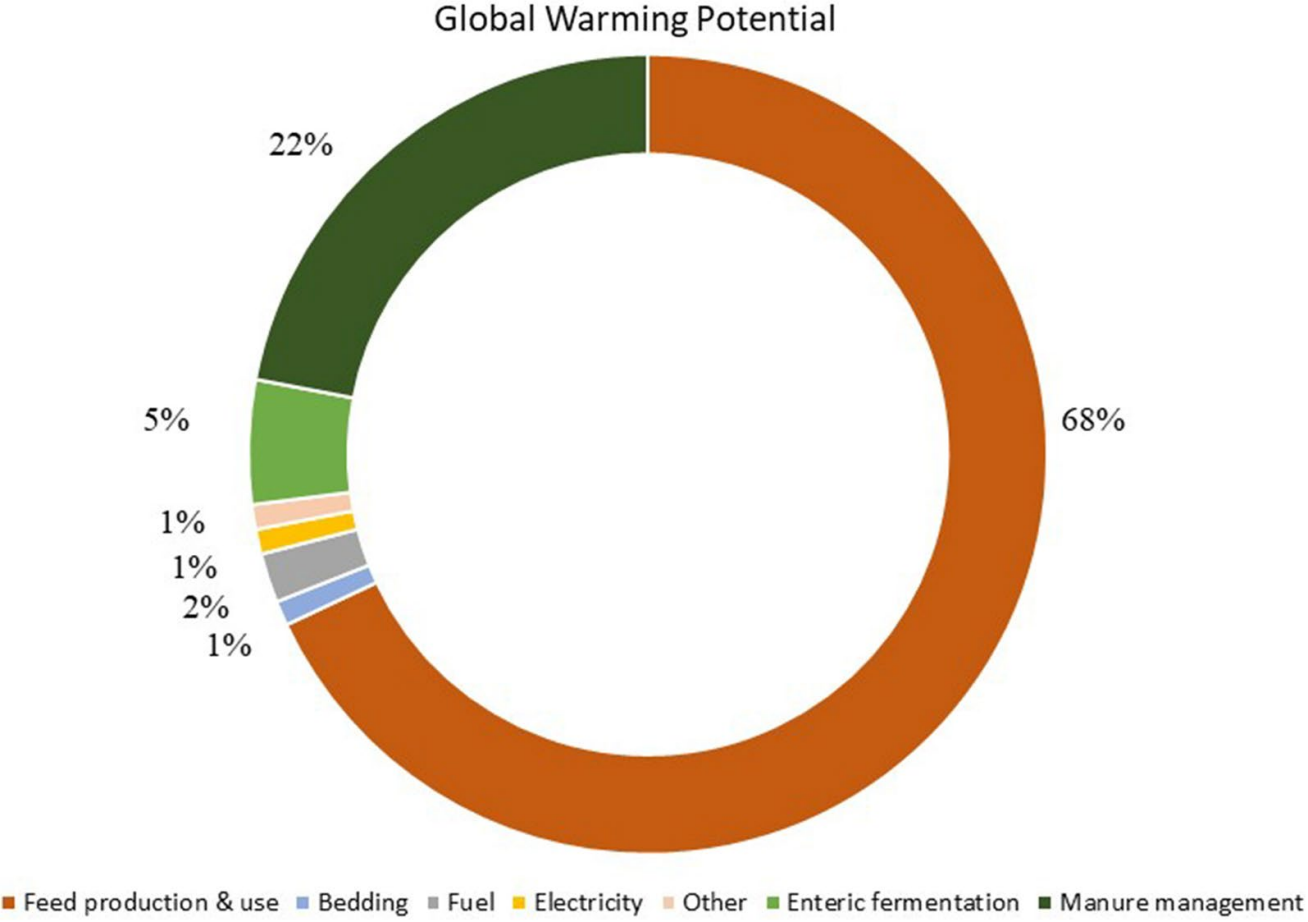
Key system components, processes, and inputs associated with environmental impact hotspots at industrial pig production systems. The estimates here are associated with the Global Warming Potential of the pig production system. Adapted from [15]

A wealth of studies has also focused on various system points, processes and components where there is a higher probability that these impacts may arise from and the pathways through which they may threaten whole system sustainability; such system points are defined as *“environmental impact hotspots”* [81, 87]. Despite the lower potential impacts when compared to other livestock systems, the complex configuration of industrial pig systems involves several production components that make extensive use of resources. These include among others, (i) fertilisers and pesticides for feed production, (ii) energy for heating, lighting, and fossil fuel for on-farm operations and transportation, (iii) water for animal growth, crop irrigation, and cleaning, (iv) waste management, and (v) land use and land transformation [79]. To better understand the environmental consequences of such processes and resources for pig production, as well as the effectiveness of potential solutions that aim to mitigate those, literature has long suggested that a whole-farm perspective should be adopted to consider all different system components, impact trade-offs, and alternative scenarios (e.g., management practices, system configurations, production efficiencies) [80]. Considering this, studies have used cradle-to-farm gate LCA to evaluate pig system environmental performance, a systematic modelling approach that considers broad system boundaries and detailed modelling of the system components and their interactions (Fig. 2). LCA studies have shown that feed production and especially soybean meal production, along with manure management account for more than 85% of GHG emissions arising from the operation of pig systems globally [2, 65, 87, 98]. When considering overall pig system environmental impact, feed production is identified as the largest contributor (~ 70%), followed by manure management (~ 20%), direct emissions from animals (~ 5%), energy use (~ 3%), and other inputs/processes (~ 2%) (Fig. 3) [15, 73, 79, 98]. Analyses of the different developmental stages involved in conventional pig production across the globe, found that the finishing stage (approximately 65 kg bodyweight to slaughter-weight) is the largest contributor to pig system environmental impact [92]. Studies in European pig production have found that a pig fattening unit is associated with up to 10 times higher environmental impacts than a weaning unit for all impact categories assessed [90, 92]. This is potentially due to that the period of growing and fattening of pigs is significantly longer compared to other life stages (e.g., lactation), due to the amount of feed consumed during this period to gain the required weight for slaughtering, and the amount of manure produced [98, 110, 129].

Due to their significant contribution across all impact categories discussed above, namely LU, GWP, AP, EP, impacts to biodiversity, and WRU, the review focuses on feed production and manure management as the two most critical environmental impact hotspots for indoor conventional pig production, and areas that can be potentially improved through existing solutions. However, it adopts a holistic perspective acknowledging the significance of interactions between various system components as well as of potential bottlenecks and trade-offs in the implementation of proposed solutions.

### Literature review methodology

The focus of our approach was exclusively on pork production. The aim of this study was to review exhaustively and summarise information sourced from scientific and grey literature regarding the broad theme of identifying environmental impact hotspots associated with European pig production systems, and bottlenecks to the implementation of potential solutions that could enhance sustainability of pig systems. Although we did not conduct a systematic review we searched for terms that were associated with feed production, animal growth, and manure management components of the pig production system as key environmental impact hotspots with potential to be improved from an environmental perspective.

To facilitate reviewing of scientific literature we used the Web of Science, Scopus, and Google Scholar search engines. Further to these, we used Google to source grey literature i.e., governmental reports, white papers, and industrial technical reports among others.

For our review, we considered studies published after 2010 and up to 2023 (year of submission). We did not narrow the focus of our search to specific emissions or impact categories, but rather explored all literature that included any of the terms presented in the Table 1.

**Table 1:** Search criteria and terms used to facilitate the scientific and grey literature review

Environmental implications	Production system	System component
Broad search at *environmental impact* level incl., any potential *impact categories* and *pollutants* i.e., no specific exclusion criteria here	Exclusive focus on *pig systems* also referred to as *pork* or *swine production systems*	*Production* (i.e., whole-farm)
		*Feed* (incl., production, use, strategies, diet formulations)
		*Animal growth & performance* (incl., pig, swine genetics, health & welfare)
		*Manure* (incl., management, storage, treatment within and beyond farm gate)

While our focus was exclusively on pig production systems, we did not limit our search to specific criteria relating to potential environmental implications, and investigated impacts and potential for abatement of those across different system components

While we focused our discussion on potential solutions to support European pig production, we did not limit our search based on geographical or system specific criteria (i.e., indoors intensive pig systems only) and therefore, sourced relevant data and information from pig systems globally that could potentially be adopted by the European pig sector.

### Feed production as an environmental impact hotspot

Feed production is a very resource intensive component in pig production that requires vast areas of land and large amounts fossil energy and water, thereby being associated with severe negative environmental implications [13, 73]. The fact that conventional pig systems source significant amounts of feed ingredients (e.g., soy) through a globalised network (i.e., via international and intercontinental trading), often leads to a disconnection between the production of feed with its use and to underestimates of the pig system environmental impacts that are virtually shared between several countries [2]. Annual European pig production requires approximately 18.4 million tonnes of soybean imported from Latin America, which occupies close to 14 million hectares of cropland outside the EU [61]. Sporchia et al. [118] specifically found that EU pig production relies heavily on resources from Brazil, Ukraine and the US to cover its pig feed needs. They also estimated that wheat-based feeds account for 32% of the land required for pig feed production, followed by soy (15%); however, due to location of production, the land-use related impacts associated with soy may outweigh the ones for other pig feeds in terms of severity and urgency to address. Most of the potential environmental impacts of different conventional feed crops are comparable in terms of urgency to address (e.g., land-use and land-use change, GHG emissions), however soy is widely viewed as the most damaging crops and the expansion of soy production to support livestock has been associated with increased land degradation and deforestation in endangered ecosystems and biodiversity hotspots globally, especially the Brazilian tropical savanna (Cerrado); this is why soy is in the core focus of this review when discussing impacts associated with the feed production component [51, 61, 116]. Land-use related impacts are in turn associated with GHG emissions, as soil transformation and processing increases the release of potent gases like CO_2_ and N_2_O; due to such disturbances to the soil and heavy reliance on synthetic nitrogen fertilisers, feed production is among the largest contributors to N_2_O [13, 57]. Furthermore, expanding the soy production sector in global South has led to increased biomass burning for deforestation, largely contributing to anthropogenic climate change and atmospheric pollution [52]. Such impacts on South American rainforests are significant drivers for species extinction and water stress, which has put a lot of pressure on EU policy makers to explore soy alternatives and develop governance initiatives to reduce reliance on soy imports from deforested areas [51, 82, 113]. In Chinese pig production too, one of the largest producers of pork meat, approximately 20% of total Chinese cropland (i.e., ~ 25.5 Mha) is used for the production of pig feed (mainly corn and wheat) and approximately 23 million tonnes of soybean are imported from countries of Latin America (e.g., Brazil, Argentina) annually for pig production [123]. For means of comparison, soy production takes up 4.6 Mha only in the Brazilian Amazon and is rapidly expanding [116]. The Centre of Innovation Excellence in Livestock (CIEL) recently published a study that highlights the significant negative impacts of land use change for pig feed production purposes, among other livestock systems, on carbon sequestration and the reservoirs of organic carbon in UK soils [15].

Aside from land use related environmental pressures, the operation of machinery for field operations (~ 63% of total energy-use) and the production and use of synthetic fertilisers, herbicides and pesticides (~ 37% of total energy-use) are significant contributors to GHG emissions, energy and fossil fuel depletion [53, 144]. Furthermore, the large, globalised network supporting pig feed production and supply significantly contributes to GWP and fossil energy depletion through emissions and resources requirements associated with the transportation of large amounts of feed over very long distances [79, 102]. Overall, feed production is responsible for almost 70% of GWP associated with pig production (Fig. 3), and with damaging crops such as soy making up as much as 20% of pig diets it is important to consider potential alternatives that could improve pig feed sustainability [102]. Such potential solutions could be landless and circular alternative feed ingredients and protein feed sources (e.g., food waste, bakery and confectionery by-products), or novel management practices at crop production that could enhance efficiency and environmental performance of field operations (e.g., conservation tillage, pre-plant fertilisation) [102, 114]. A requirement for more intense pig production will undoubtedly worsen the environmental impacts associated with pig feed production. Further to the direct environmental impacts discussed above, there is an increasing concern for a “feed versus food versus bioenergy” competition for resources, mainly land and water, which further suggests an urgent need for feed ingredient diversification [85].

### Manure management as an environmental impact hotspot

Pig manure management is the second largest contributor to pig system environmental impact, accounting for 18% of total GHG emissions associated with the livestock industry globally [26]. Literature suggests that CO_2_, N_2_O, and CH_4_ are the main GHG emissions associated with pig manure excretion, management, storage, and application [98, 103]. Emissions from this system component and their associated impacts are directly affected by specific properties of pig feeds (e.g., crude fibre levels) and pig performance traits (e.g., nutrient retention rates at various life stages). For example, CH_4_ and CO_2_ emissions from manure are positively related to the concentration of fibre, organic matter, and specifically volatile fatty acids in the animals’ diets. Nitrous oxide is principally related to the amount of nitrogen in pig feeds (i.e., crude protein levels), but its formation is a result of incomplete denitrification processes at manure storage and management (i.e., after excretion) and is increased in the presence of oxygen e.g., when a large surface of the manure is in direct contact with air [103]. For this reason, when evaluating pig system environmental impacts, it is important to follow the nutrient and emission flows down the “feed * animal * manure” pathway. This approach is particularly useful for N and P related emissions, which can be significantly reduced by enhancing nutrient utilisation or modifying diet compositions to reduce nutrient content, as discussed previously. When considering carbon emissions in the livestock sector more generally, a lot of discussion has focused mainly on methane from enteric fermentation in ruminants. In pigs however, due to the different digestive system (i.e., monogastric) and significantly less dietary fibre involved, it is estimated that 78% of carbon emissions arise from pig slurry and only 22% directly from the animal; enteric fermentation comprises 4–5% of the latter while the rest is accounted for by pig respiration [103, 110]. Therefore, when seeking solutions to improve pig system environmental performance it is critical to look also beyond enhancing pig genotypes or modifying diets, and into the specific manure management and treatment practices.

Manure management is a complex system component that involves several processes which all contribute to pig system environmental burdens—some more significantly than others. The three main parts that manure management consists of are: (i) in-house storage and treatment, (ii) outside storage and treatment, and (iii) application of treated or untreated manure at crop production as organic fertiliser [26, 71]. The main sources of environmental impact across the manure management chain that need to be controlled and mitigated if possible are CH_4_, NH_3_ or total ammoniacal nitrogen (TAN), and total N and P concentrations [56].

At conventional pig systems, manure is stored as slurry in pits located underneath the pig housing system. In addition to the physical and chemical properties of slurry that are largely defined by the feed composition and nutrient retention rates at different pig life stages (e.g., concentration of nutrients and volatile solids, urine: faeces ratio), several external factors may significantly affect environmentally harmful emissions. For example, ammonia emissions from pig slurry can be significantly increased due to the infrequent removal of slurry pens and slurry pits increases potential ammonia emissions, high temperatures and low wind speeds at pen and slurry pit levels, and with more surface of slurry being exposed and in contact with air [56, 112].

During outside storage, the type of facilities plays a crucial role to overall emissions from pig manure management, with large covered slurry tanks considered as a good baseline. At this stage, variations in the duration of storage (usually several months long), amount of dilution due to rainfalls, and climate conditions can significantly affect relevant emissions [1, 58, 98]. Finally, slurry is applied at crop production as an organic substitute to synthetic fertilisers. While these emissions are typically accounted for within the feed production system component, it is important to discuss them here and highlight the circularity that characterises nutrient flows throughout the pig production system, especially relating to nitrogen concentration and emissions as this is an important input in crop production (e.g., use of ammonia and urea fertilisers); this circular pathway suggests the need for holistic assessment of any decisions made at the different pig system components e.g., changes in feed composition→changes in manure composition→altered properties of manure as fertiliser→varying emissions at pig system level. At manure application, emissions can be affected by climate conditions, geomorphology, and crop characteristics; a combination of heavy rainfalls, inclined fields, and coarse-textured soils can greatly increase nutrient leaching from soils to nearby water bodies increasing risks for eutrophication and deterioration of water quality [63, 98].

## Solutions towards pig system environmental sustainability: opportunities and bottlenecks

**Fig. 4:**
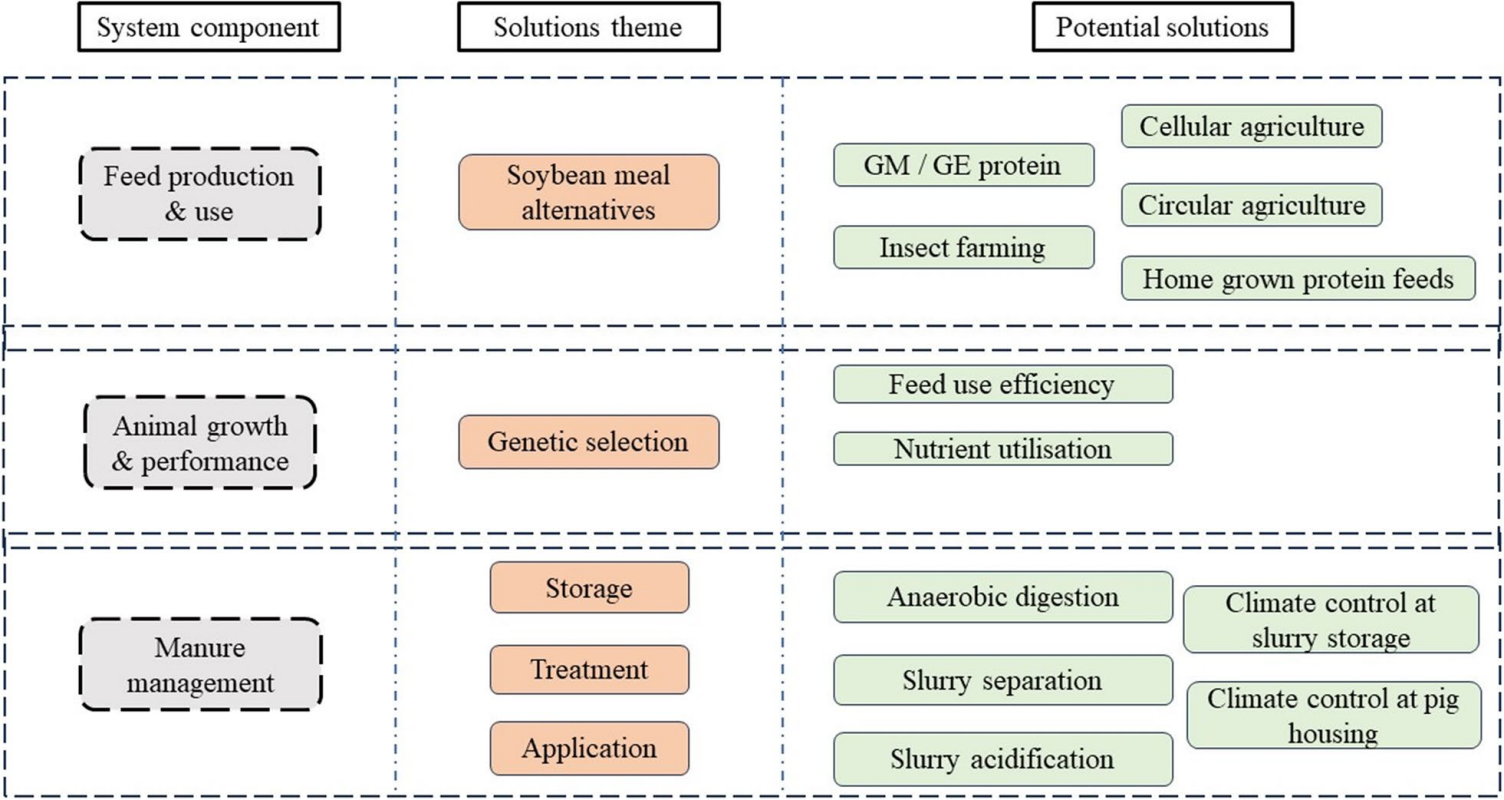
Summary of potential solutions towards improving pig system environmental sustainability considering management practices and technological innovations in the feed production & use, animal growth & performance, and manure management system components

Over the years, several management practices and technologies have been developed and implemented at various pig production scales aiming to reduce pig system environmental impacts at specific production chain hotspots. Despite the amount of research invested in identifying optimal solutions for system productivity and environmental sustainability, the implementation of such potential solutions is often hindered or slowed down by internal or external to the pig system factors—defined as *bottlenecks*. Using the recent CIEL expert opinion report [16] on how pig production farmers can mitigate emissions arising by the various system components as a starting point, we followed the same approach and reviewed in more detail three key aspects of the pig production system that have potential to enhance system sustainability: (i) the animal, (ii) the feed, and (iii) the manure management (Fig. 4). While adopting a similar perspective with [16], this study reviews the important interactions and overlaps between the three system components throughout (e.g., the need for efficient indoor climate control to ensure pig health and welfare, and reduce emissions from pig slurry) and discusses specific bottlenecks that hinder implementation of the alternatives, thereby enabling future efforts to overcome them.

### Animal health and genetic selection for enhanced feed use efficiency and nutrient utilisation

Until recently, the pig breeding and genetic selection focused mostly on improving animal performance to maximise production, and so introduced animals with (i) enhanced feed use efficiency, nutrient utilisation, and pig growth rates at different developmental stages (11.5–14.5% improved feed conversion rates), (ii) improved reproductive performance (i.e., gestating and lactating sows), and (iii) reduced mortality rates, while improving meat quality among other carcass traits [93, 141]. In doing so however, it became evident that genetic selection can provide a potential solution to reducing pig system environmental impact without sacrificing farm profitability and product acceptability [141]. Indeed, research on historic trends of pig traits and environmental impacts of pig production in Great Britain revealed significant reductions over the years as animal performance improved due to selective breeding, across several impact categories [93]. The specific findings showed that GWP was affected the most, potentially due to reductions over time of environmentally damaging feed ingredients in pig diets, for example, more efficient genotypes can achieve optimal animal growth with lower inclusion levels of soy in their diets. While these studies focused on soy substitutions specifically, their findings reveal the potential for other less balanced protein sources (e.g., sunflower meals, fishmeals, rapeseed meals) to be replaced or partially substituted by more sustainable alternatives. Lower crude protein levels in pig diets in combination with improved utilisation of N and P from enhanced genotypes (i.e., less N and P excreted), highlights further potential for significant mitigation of AP and EP arising from pig systems; Zhang et al. [141] found that transgenic pigs exhibit better N and P utilisation by 23.2–45.8%.

While the search for new and improved genotypes to breed for reduced pig system environmental performance has potential to progress further with advancements in biotechnology, scaling up pig production can present challenging conditions for animal health and welfare which bottleneck the efforts for environmentally sustainable and resilient pig production [50, 133]. Tallentire et al. [121] have questioned the feasibility of further improvements in feed utilisation efficiency of livestock and the consequent environmental performance of their systems, without compromising other components of the system, which actually contribute negative on the latter. Even for enhanced genotypes, external factors can lead to impaired animal health and welfare and consequently to detrimental effects on system environmental performance due to higher feed conversion ratios, and issues of nutrient utilisation and feed digestibility among others. Such external factors can be linked to infections and diseases (i.e., pathogens, bacteria, viruses) and can be caused due to poor farm management practices for example, improper feed storage, infrequent waste removal, overcrowding of pens. Therefore to avoid such risks, good farm management practices and frequent biosecurity assessments should be implemented, including proper maintenance of feed and water storage and supply equipment, thorough cleaning and disinfection plans, and herd management sufficient provision of bedding and enrichment to limit aggressive behaviours and occurrence of vice [5, 25]. Approximately 5.9% lower CO_2_ emissions at pig system level have between healthy and impaired fattening pigs have been estimated [9], although the exact quantification of the contribution of impaired health and welfare to the environmental impact of livestock, and pig systems in particular, is still pending [74].

Another common management practice to combating potential risks to animal health, is the administration of antimicrobials. While this may be effective in ensuring animal growth, studies have shown that up to 90% of the administered antimicrobials are excreted through manure [66]; this can lead to significant issues for environmental sustainability and human health as manure is applied to fields for crop production and therefore, traces of antibiotics and their metabolites are absorbed by plants via uptake, eventually reaching the pig feed and human food chains. Accumulation of antimicrobials in groundwaters and soils can further lead to transformations of the microbial biodiversity (e.g., enhance resistances of bacteria), largely reshaping soil and aquatic ecosystems [139]. Consequently, considering such significant trade-offs, a holistic approach for good feed, herd, and manure management practices is required to ensure both pig system productivity and environmental sustainability.

Besides poor farm management practices, climate change can exacerbate risks for potential pathogen outbreaks, for example mycotoxin contaminations through feed [88]. Prolonged hot and humid climate conditions can cause heat stress to pigs especially at indoor intensive systems, which also has direct negative implications on their feed intake (− 17.2%) and growth rates (− 38.7%) for the duration of the thermal discomfort period [49]. Chronic or acute heat stress can lead to significantly increased respiration rates with experiments having identified changes from 40 breaths per minute under normal conditions to up to 85 and 125 breaths per minute (chronic and acute heat stress respectively) [48, 132). Considering the CO_2_ emissions associated with respiration at different life stages (e.g., 1.70 kg CO_2_ per day per fattening pig), such increases can contribute negatively to system Carbon footprint particularly in larger production systems (i.e., tens of thousands of fattening pigs) [103]. Decreasing pig feed intake in periods of heat stress and modifying diet formulations can help mitigate adverse effects of increased temperature and humidity. Specifically, studies found that reducing crude protein and increasing fat levels improves average daily gains of fattening pigs during heat stress, since the heat increment for crude protein metabolism is significantly higher than that of fats [6, 111]. Effective indoor climate control regulation is another critical requirement to mitigate impacts of heat stress on pig system productivity and environmental performance, which entails among others pig cooling and showering systems, and well-ventilated and well-insulated infrastructure for stable indoor temperatures regardless of ambient conditions [48, 77, 100, 101].

### Alternative feed ingredients and feeding strategies

Due to the great significance of the feed production component in pig system environmental performance, investigating alternative feed ingredients, diet formulations, and precision feeding strategies has long been in the centre of research aiming to improve livestock system sustainability [73, 123]. Precision feeding, which involves the collection and analysis of data on animal performance to inform proper nutrient provision through suitable diet compositions, can help reduce N and P excretions by close to 40% and pig system GHG emissions by up to 6% [106]. While significant abatement potential can be achieved through precision pig farming approaches, there is still a need to make feed production more sustainable. As discussed previously, the production and importation of protein feeds, especially soy, are associated with severe threats to the pig sector environmental sustainability. More than 50% of global soy production that is intended for livestock feeds takes place in South America, where it is associated with deforestation, land degradation, and negative impacts on biodiversity of the Amazon rainforest [61]. Prior to the Russia—Ukraine conflict, reports suggested that a significant expansion of Ukraine’s agricultural sector through EU investments could increase oilseed production (mainly soybean, sunflower, and rapeseed) by up to − 84% therefore reducing reliance of EU pig production on soy imports [27]. However, the latest geo-political developments forced a freezing of the relevant EU investments and disrupted supply/exports of Ukrainian oilseeds, cereals, and chemical inputs that conventional pig feed production relied on, to EU and other countries [102].

To tackle the concerning environmental impacts associated with conventional pig feed production and enhance its resilience to extreme events, review and stakeholder engagement studies have identified pig feed alternatives along three key directions: (i) Local, (ii) Landless, and (iii) Circular feed ingredients and production methods [102]. While there can be large overlaps when attempting to categorise potential pig feed alternatives as “local or landless or circular”, we do so by focusing on the characteristic of the feed alternative that allows it to generate the most significant environmental benefits. For example, food waste could be considered at the same time as a local (i.e., sourced from local households or retailers), a landless (i.e., no land requirements associated with its intended use as pig feed), and a circular alternative (i.e., upscaling of wastes); we consider “circularity” to be its most defining and representative characteristic for the context of this review, because it fully encompasses its potential to reduce a greater range of environmental impacts compared to other local or landless alternatives [102].

A recent report published by the Food Standards Agency (FSA) of the UK [102] and extensive UK stakeholder workshops facilitated by Queen’s University Belfast in association with CIEL, have further identified key criteria that potential alternative feeds need to fulfil to enable their commercial implementation by pig systems. Such criteria are the need for: (i) continuous and consistent supply, (ii) consistent nutritional profiles, (iii) palatability and animal acceptability, and (iii) feed and food safety. They have also identified potential barriers that future research may help overcome to further facilitate large-scale production and incorporation of alternative feeds, such as: (i) enhancing the availability of information regarding variability in nutritional profiles and implications on animal performance, (ii) enhancing consumer acceptance, and (iii) lowering cost of production and implementation to levels comparable with conventional feeds.

### Local feed ingredients

Local feed ingredients and production methods primarily aim to mitigate some of the more urgent environmental issues of feed production and supply, that are linked to the expansion of cropland in forested areas and biodiversity hotpots, and the emissions arising from the transportation of feeds over long distances.

#### Home grown feed ingredients

A large body of research, particularly in Europe due to its reliance on imported soy, has investigated the substitution of soy and conventional cereal crops with local feed ingredients for example, lupin, peas, fava beans, alfalfa, clover, and duckweed [109, 115, 135]. Van Zanten et al. [130] found that replacing soy with locally grown rapeseed in fattening pig feeds can potentially reduce land use up to 12%. Future climate change projections suggest that areas of the global North (e.g., Canada, Northern Europe) can even become suitable for large-scale production of soy, thereby relieving significant pressures such as deforestation and biodiversity loss in sensitive ecosystems of the global South (e.g., forest ecosystems of Brazil and Argentina) and enhancing resilience of global pig production [21, 54]. This evidence for large and healthy yields of conventional feeds in the North, in association with the potential for more diverse local sources to be incorporated in pig diets, suggests that requirement for uninterrupted and consistent supply of feeds could be met. In addition to this, enriching feed options with “new” local crops can increase overall pig system resilience to extreme events, like the significant delays and disruptions in feed availability caused by the Covid-19 pandemic, Brexit, the Ukraine—Russia conflict, and animal disease outbreaks in large feed exporters of the globe. However, when exploring the commercial implementation of home grown alternative crops, it is important to consider potential knock-on effects. These may include land-use change and soil transformation related impacts e.g., increased GHG emissions due to field operations in the North, invasiveness of new species e.g., in the case of genetically modified / edited genotypes for feed production as will be discussed below, and competition for resources namely land, energy, and water against established industries of the global North e.g., biofuel, cereal crop production [102].

Further, to reducing globalised environmental burdens, utilising home-grown feed ingredients presents an opportunity to significantly reduce pig system GWP by eliminating the need for long distance transportation of very heavy hauls.

However, there are also several barriers and potential risks associated with growing local crops to scale i.e., to adequately support livestock production by substituting more environmentally impacting feeds. Spatially shifting feed production may leave large areas of current cropland mismanaged and abandoned. The restoration of such degraded land into healthy soils, soil sequestration processes, and organic carbon stocks can be a very challenging task, especially considering the increased frequency of extreme climate phenomena such as prolonged heat waves and droughts that can worsen such environmental impacts [78, 136]. From an animal performance perspective, the implementation of local alternatives can be facilitated by overcoming barriers in the understanding of how variable the nutritional profiles of such feeds are, and by a wealth of knowledge around the impact of different inclusion levels or feed combinations/formulations on animal performance, health and welfare, and meat quality (e.g., impacts of lupin antinutritional factors) [24, 86].

#### Genetically modified and engineered feed ingredients

Genetically modified and engineered (GM/GE) plant crops have been a popular alternative to conventional crop types that has been commercially implemented in pig feeds for several years. The most popular of these have been GM/GE types for soy, maize, and potato. GM soy Estimates suggest that 70–90% of the total GM/GE crop biomass globally is consumed by livestock [102]. The benefits of using GM/GE crops arise mainly from their enhanced resistances to pests, weeds, plant diseases, water stress, and climate conditions. For example, maize and potatoes that have been modified with the *Bacillus thuringiensis* (*Bt* variation) gene produce the Bt toxin, thereby being resistant to insects and returning a higher yield [4]. Other potatoes (*AmA1* variation) have been modified to express up to 40% more protein than the conventional cultivated or wild genotypes [127]. Soy has been modified in various ways in the past, with some phenotypes producing enhanced nutritional quality (*Glycine max* variation), others resistances to glyphosate (*Roundup Ready* variation), and others resistances to drought (*Drb2a/Drb2b* variations) among other improved characteristics [4]. These crop properties can help reduce environmental impacts associated with the production and use of chemical and synthetic inputs including reduced GWP, fossil fuel use, EP, negative impacts on biodiversity, and overall pig system water footprint, while reducing overall production costs and often enhancing yield [47]. While GM / GE crops can be cultivated in a great range of geographies both in the North and South hemisphere, their enhanced phenotypes highlight their potential to facilitate a shift of in feed production to less suitable areas of the globe (e.g., North) that however satisfy other important criteria to improving pig system sustainability (e.g., less transportation to pig farm for lower emissions and costs) [102]. The review discusses GM/GE crops as a local alternative to emphasise this potential.

Despite the many environmental benefits that GM/GE feeds may offer, important bottlenecks need to be overcome for them to satisfy the criteria for safe and viable incorporation in pig feeds previously mentioned. From an environmental perspective, when considering GM/GE feed production, mechanisms should be in place to mitigate the potential land use related impacts discussed above and potential negative impacts to biodiversity due to gene flow/gene transfer, weediness (i.e., expansion of enhanced genotypes beyond the intended boundaries), and increased use of chemicals to combat such resistant weeds (i.e., chemical inputs could lead to severe EP impacts in near freshwater and coastal ecosystems) [35].

In relation to animal health, literature suggests that more research is required to safely draw conclusions about GM/GE effects on animal growth rates, gut intestinal health, and organ function. While studies using haematology blood tests on pigs fed specific GM feeds (e.g., Bt MON810 maize) have found statistically significant changes in serum biochemical parameters, such changes fall within normal reference values and therefore more long-term experiments (especially using second generation GM crops) are required to trace potential changes on potentially vulnerable organs like the liver or kidneys [28, 29].

At consumer level, the debate and misconceptions regarding the use of GM/GEs that reach the human food chain either directly or through bioaccumulation present a perpetuating barrier. Further information from credible sources, towards both producers and consumers, regarding the potential sustainability trade-offs associated with such alternatives may eventually help increase consumer acceptance and enable their commercial implementation at pig production [46, 60, 62].

#### Insect farming

Insect meals contain between 50 and 82% crude protein as well as other important nutrients (e.g., fatty acids, calcium, iron) that promote good gut health and nutrient utilisation [75]. Therefore, incorporating insect meals in pig feeds as a protein source may improve system environmental performance both through reducing reliance on unsustainable soy and through improving pig feed efficiency i.e., less N and P excreted in manure results to reduced AP and EP impacts which is critical particularly when manure is applied near N and P vulnerable zones [91]. Depending on the substrate used to rear insects (e.g., using industry by-products and wastes) generated from the pig system, this alternative can also help mitigate impacts associated with waste processing and disposal. Currently only human in-edible food waste and former foodstuffs are used for commercial rearing of insects, due to strict EU and US policies that aim to prevent disease outbreaks and bioaccumulation of chemical contaminants in the feed and food chains [12]. However, several studies have explored the potential benefits of circular streams that upscale either untreated pig manure or the digestate from the anaerobic co-digestion of manure with other household and industry wastes, to rear insects for pig feeds [10]. Specific findings of such studies highlight that insects can convert up to 13% of NH_3_ from manure and digestate to body growth, therefore significantly reducing N related unintended impacts at manure storage and field application [104]. Another benefit of insect farming is that it does not require any synthetic fertilisers and chemical inputs, the production and use of which significantly contribute to fossil energy depletion, GWP, AP and EP impacts [134].

Despite the potential environmental benefits that insect farming for the pig production sector, it is important to consider that when implemented at commercial scales it may be presented with several bottlenecks including: (i) knock-on effects on land transformation from the potential displacement of other agricultural activities i.e., insect farms are not landless units and therefore mass rearing may drive significant land-use change [32], and (ii) increased GHG emissions associated with large energy requirements for climate control at insect farms particularly if this energy is not obtained from renewable sources [8].

Insect farming is still in its infancy and therefore, it is likely that a number of issues associated with mass insect rearing for animal feed, including the ones mentioned above, need to be addressed before the practice has widespread application.

### Landless feed production methods

Even though local solutions can help disentangle pig feed production from deforestation and land pressures in sensitive ecosystems, some landless alternatives can further help improve pig feed environmental sustainability by considering also potential knock-on effects of land use and displacement of local biodiversity caused by local feed production.

#### Protein sources from cellular agriculture

Cellular agriculture refers to the production of agricultural products, in this case pig protein feeds, from cell cultures using biotechnology and associated methods and techniques [94]. Throughout the years, advancements in biotechnology have opened new ways of incorporating high-quality protein feeds from micro-algae, fungi, and bacteria, as a substitute or dietary supplement to conventional feed ingredients. Many of these alternatives gained significant popularity (e.g., yeast protein, micro-algae, protein from methane-utilising bacteria) and studies have investigated their potential nutritional benefits in detail. From an environmental perspective, recent technological developments have introduced the potential for microbial protein to be cultivated on waste substrates (e.g., food waste), therefore significantly improving the environmental performance of pig production in addition to nutrient provision [125]. Microbial protein requires significantly smaller production and supply systems and lower energy than conventional protein feed production, therefore presenting large mitigation potential for unintended LU, GWP, and NRRU impacts associated with pig feed production [6]. Potential synergies between cellular agriculture and circular pathways, for example using pig manure or former foods and food waste to produce cell protein, highlight further opportunities towards reducing environmental impacts associated with manure application as fertiliser or waste disposal [7, 70, 96, 143].

However, it is important to consider that the performance of cell protein extraction processes can be significantly affected by the choice of waste substrate, production conditions (e.g., pH, temperature) and potential antagonistic effects between various microorganisms used at production. For example, studies have found that protein content can range between 5.7% and 71% depending on the type of waste and microorganism of choice, while pH and temperature during hydrolysis can affect protein yields by approximately 50% and 15% respectively [70, 117, 143]. Therefore, this high sensitivity may present challenges in ensuring consistent provision of nutrients for pig growth using cellular alternatives. Another key bottleneck to advancing protein feed production through cellular agriculture is the rate of development for technologies that will: (i) help streamline and bring the process to large-scales, (ii) monitor and detect potential chemical and biological contaminations particularly when waste substrates are used, and (iii) improve palatability and texture of microbial protein feeds to enhance animal acceptability. Furthermore, enhancing current knowledge through additional research on the interactions between waste substrate, nutritional profile and efficiency of cell feeds, and animal performance, is important to allow pig producers to use such alternatives more effectively and target whole system sustainability.

#### Landless crop growing methods and feed supplementation

A popular alternative plant-based pig feed, grown in a rather unconventional way considering traditional feed ingredients, is seaweed (macroalgae). Although the protein content of seaweed can vary between different species (e.g., *Porphyra sp.*, *Fucus vesiculosus*, *Palmaria palmata*) from 3 to 47%, literature suggests that they offer a suitable nutritional profile for inclusion in pig feeds, also providing a wide range of vitamins, minerals, and fatty-acids [22, 84].

Hydroponic fodder from cereal grain (e.g., barley, maize, triticale) present another potential solution to substituting conventional protein sources in pig feeds. This alternative cultivation method could yield traditional feed ingredients with properties that potentially improve nutrient digestibility and animal performance, while generating environmental benefits by reducing the land footprint and reliance on synthetic inputs (e.g., fertilisers) of conventional feed production [76]. Further environmental opportunities of hydroponics can be unlocked when wastewater is upcycled to input for production [18].

Amino acid supplementation is a well-established feeding practice especially at earlier pig life stages, and is commonly used to improve N use efficiency since synthetic amino acids can provide balanced dietary protein for pig growth, while also resulting to improved meat quality and texture [72]. Studies on pig system environmental impacts under scenarios where crude protein content of diets is reduced with amino acid supplementation and at constant nutrient retention rates (therefore less total N excreted with manure), have shown significant mitigation potential for GHG emissions (~ 5% lower), AP, and EU (~ 28% lower) [43, 79]. Besides synthetic amino acid supplementation, it is important to note that feed additives such as acidifiers, phytases, proteases and multienzymes have long been supplemented in pig diets to help improve animal performance through regulating gut microbiota and boosting intestinal health and immune system, as well as aiming to mitigate system environmental impact by reducing specific harmful emissions and resource use required for production [3, 68, 108].

While evidence in literature regarding the environmental benefits of the alternatives mentioned above is overwhelming, there is an important risk to bringing their production to industrial scales which is particularly relevant to the case of seaweed as pig feed. Seaweed requires large amounts of energy for processes including the harvesting and drying, hydroponics have big electricity requirements for lighting and water filtration, and finally synthetic amino acid production requires large amounts of energy for the fermentation process [33]. While such energy requirements are largely covered by electricity and therefore do not contribute greatly to fossil fuel depletion, it is important that the production of these alternatives becomes more efficient, and that availability of renewable alternative energy is enhanced to enable sustainable and cost-effective implementation at industrial pig production scales. Furthermore, as with many of the alternative pig feeds discussed in this review, more information is needed for pig nutritionists and producers to better understand the potential effects of these ingredients on animal performance, when used in different inclusion levels.

### Circular streams for feed ingredient sourcing

**Fig. 5:**
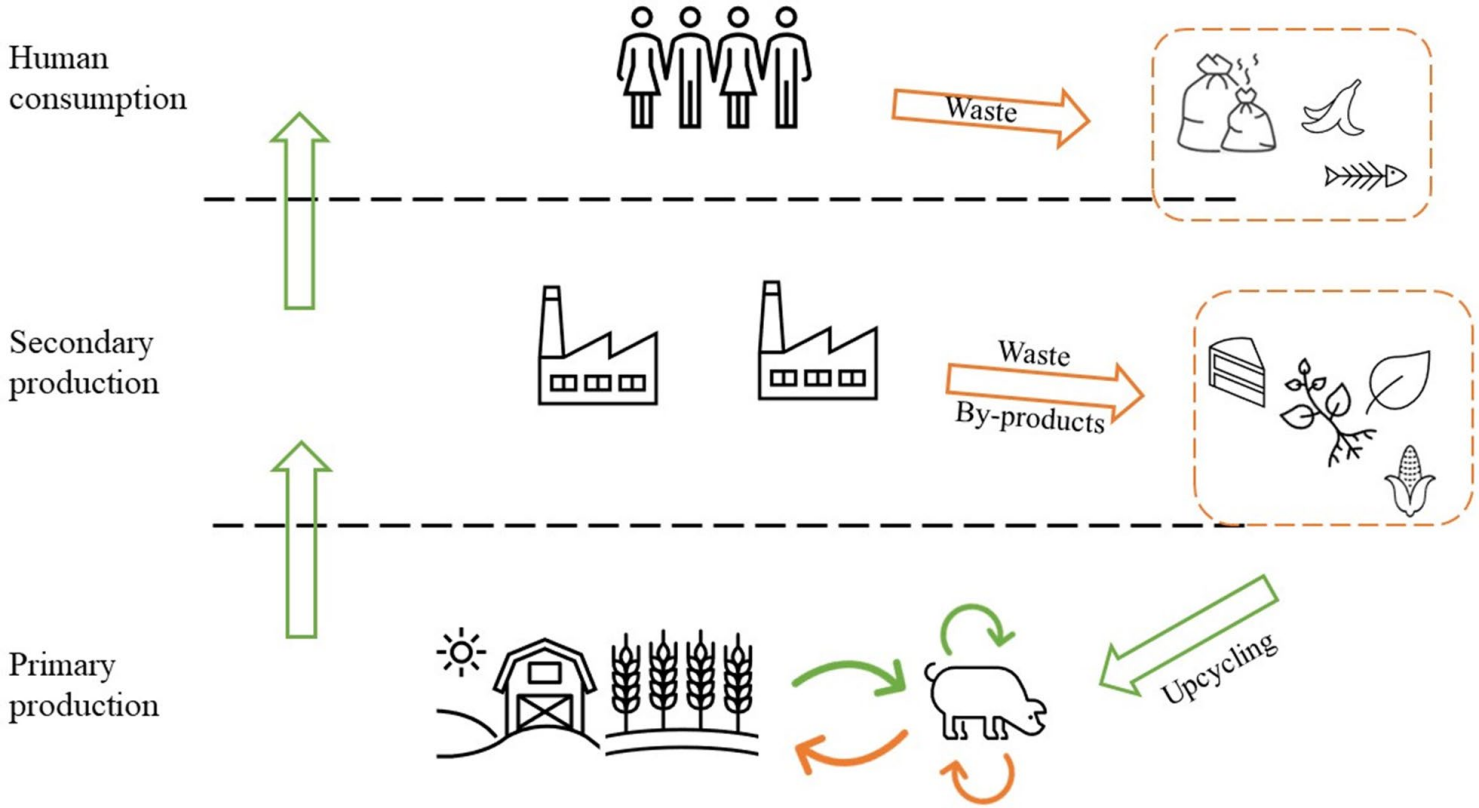
Principles of circularity and flows in the context of circular agriculture. Food waste from human consumption or industry by-productions are used as feed inputs in livestock production. Examples of waste and by-products are food waste, former foods, non-edible plant residues from crop production, by-products from biorefinery, or animal by-products

Circular agriculture is defined by practices that upscale wastes into valuable resources for agricultural production (Fig. 5). In the context of this study, we identified through literature the following circular streams that could be used for pig feed production at commercial scales: food wastes, former foods, and agricultural by-products that include animal by-products (i.e., from livestock production) and non-edible by-products of food, feed, or fuel crops. Waste streams can encompass a variety of potential feedstuffs. They include co- and by-products of various industries (e.g., hospitality) that could otherwise end in landfills, former foods and swill. Former foods must have been manufactured in full compliance with EU food safety requirements as well as the General Food Law’s demands as regards traceability, and should be unlikely to cause any health risks to humans to become eligible for feed use [36]. When foods are not intended for human consumption due to manufacturing, packaging, or logistical reasons, they need to be processed following strict quality control standards and mechanisms before use as livestock feed, however the legislation makes a specific effort to exclude them from being considered as ‘waste’ [36, 42]. Former foods can be often confused with food and catering waste—commonly known as swill—which legislation currently prohibits from being used as animal feed. The ban imposed in 2002 aimed to minimise the risk of transmission of animal diseases following cases in Europe such as outbreaks of foot and mouth disease and African swine fever. However, recent advances in biotechnology reveal the potential that precise and effective hygienic treatment of such materials may allow for a reconsideration of such restrictions [44, 145].

#### Former foods, food waste, and industry by-products

Using former foods, food waste, and agricultural by-products has been another very popular topic of debate and research when considering more sustainable pig feed alternatives. Studies have found that approximately 30% of global arable land is used to produce food that will be wasted [124]. Providing this surplus to animals, therefore, presents not only a great opportunity to reduce livestock production sector environmental footprint (i.e., impacts that would arise from feed production), but also a solution to mitigate food waste and avoid unintended impacts of waste disposal [145]. From a nutritional perspective, evidence suggests that food waste from hospitality including hotels, restaurants, and cafeterias, and households, as well as former foodstuffs from bakery and confectionery, can adequately substitute conventional pig feed protein sources and be a great source of energy for pig growth [104, 107]. Another circular stream that can supplement and improve environmental impact of pig feed production is the use of agricultural by-products from food crop production or agroforestry that are inedible by humans e.g., plant foliage, rough leafy parts of plants, fruit and vegetable waste, and others. Extracting leaf protein from such sources or supplementing these directly helps avoid the unintended use of resources and impacts associated with their disposal (e.g., biomass burning) and can provide pigs with a good source of protein, energy and fibre [120].

The most important bottleneck to adopting pig feed alternatives from food waste and industry by-products, is the development of technologies for thorough and efficient hygienic processing of wastes, and the sensitive and precise detection of potential biological or chemical contaminants. Food wastes and former foods should be thermally treated prior to incorporation in pig diets to avoid unintended risks for viral diseases and bacterial infections [37, 102, 107]. An added level of processing should assess risks for chemical contamination from packaging residues (e.g., nanoplastics) or harmful substances used for food production (e.g., pesticides, heavy metals). Further, detection of high concentrations of harmful antinutritional factors, secondary metabolites, and toxins that can significantly impair animal health should be performed to ensure safe implementation of alternative feeds from food waste and industry by-products. Another potential bottleneck to the adoption of food wastes as pig feeds may arise from a competition with other markets and alternative uses of food waste, for example the use of waste for bioenergy, heat, and biofuel production through anaerobic digestion, which may present greater opportunities towards sustainability of the food-energy nexus [59, 145]. The European Waste Framework Directive in its 5-step hierarchy prioritises waste recycling through animal feed pathways over energy recovery, thereby promoting the potential implementation of such alternative solutions for sustainable livestock production [39]. Finally, even though waste valorisation through pig feeds generates some clear benefits for the environment and society, at a consumer level it can be associated with a feeling of “disgust”. Even when it does not affect meat quality, this can present a significant barrier to pig meat marketability and therefore risk pig system profitability [102]. A strict regulatory system that monitors the production and implementation of such alternatives, as well as provision of information regarding the opportunities, risks, and misconceptions surrounding these feeding options, can help overcome such bottlenecks.

#### Processed animal proteins (PAPs) from animal by-products

Animal-based protein involves the use of by-products such as blood, fat, bones, and feathers from various animal species. These alternative protein feeds have been considered for livestock production to balance some of the drawbacks that plant-based protein feeds are associated with, such as the low protein content or presence of antinutritional factors that may reduce feed use efficiency and nutrient utilisation [31, 34]. As a circular approach, the use of processed animal proteins (PAPs) as feeds can reduce land use up to 98% and generate significantly less GHG emissions when compared to conventional protein feeds like soybean meals [102].

A potential bottleneck of this alternative at large scales, is the energy requirements for rendering and processing of PAPs [137]. A more important bottleneck, however, is that PAPs in livestock feeds have been associated with severe animal and human health issues in past years, namely the epidemic of bovine transmissible spongiform encephalopathies, and strict legislation banned them for long to prevent such incidents from reoccurring [137]. This presents a significant barrier to their commercial implementation, but recent legislative developments such as with the EU now allowing cross-species feeding of PAPs (i.e., avian by-products to pigs), highlight how important the potential environmental and nutritional benefits of such alternatives are to securing a more sustainable and resilient livestock production considering emerging climate change and feed availability challenges [38]. Still, further advances in PAP rendering technologies, and biotechnology for the monitoring and control of disease transmission are needed to avoid further bottlenecking the implementation of these alternatives.

### Manure management and pig housing

As previously discussed, several climatic and other external factors can significantly affect emissions at the various stages of manure management. At most conventional industrial pig production systems many of those external factors are defined by pig housing facilities, management practices, and technologies, which is why the review presents potential pig housing and manure management solutions to improving pig system environmental sustainability together.

#### Sustainable infrastructure and indoor climate control

Thorough studies on US Midwest pig systems, also accounting for the construction and maintenance of the infrastructure and technologies involved in pig production, have estimated that conventional system energy requirements for ventilation & heating, feed production and feed & water delivery, illumination, washing, and manure handling, measure to 28.8 MJ/kg of body weight correspond up to approximately 65 kg of CO_2_ generated per animal [23, 65]. Other studies in European pig systems have found that variability in barn dimensions of conventional pig production systems, within the ± 95 reference intervals, can increase the GWP impact associated with the pig housing system component by up to ~ 14%, due to the additional conventional construction materials required [98]. Therefore, constructing pig houses with more energy efficient facilities and technologies may present another way to improving system sustainability. However, this can be a challenging task particularly when it involves changes in older production units and may be bottlenecked by the high purchasing costs for more sustainable and durable materials (e.g., hempcrete, recycled steel and plastic, wool and straw insulation) and technologies (e.g., geothermal heating pumps, smart climate control systems). Such solutions could potentially be facilitated in the future through a programme of relevant subsidies and regulatory pressures for more sustainable pig system infrastructure.

**Fig. 6:**
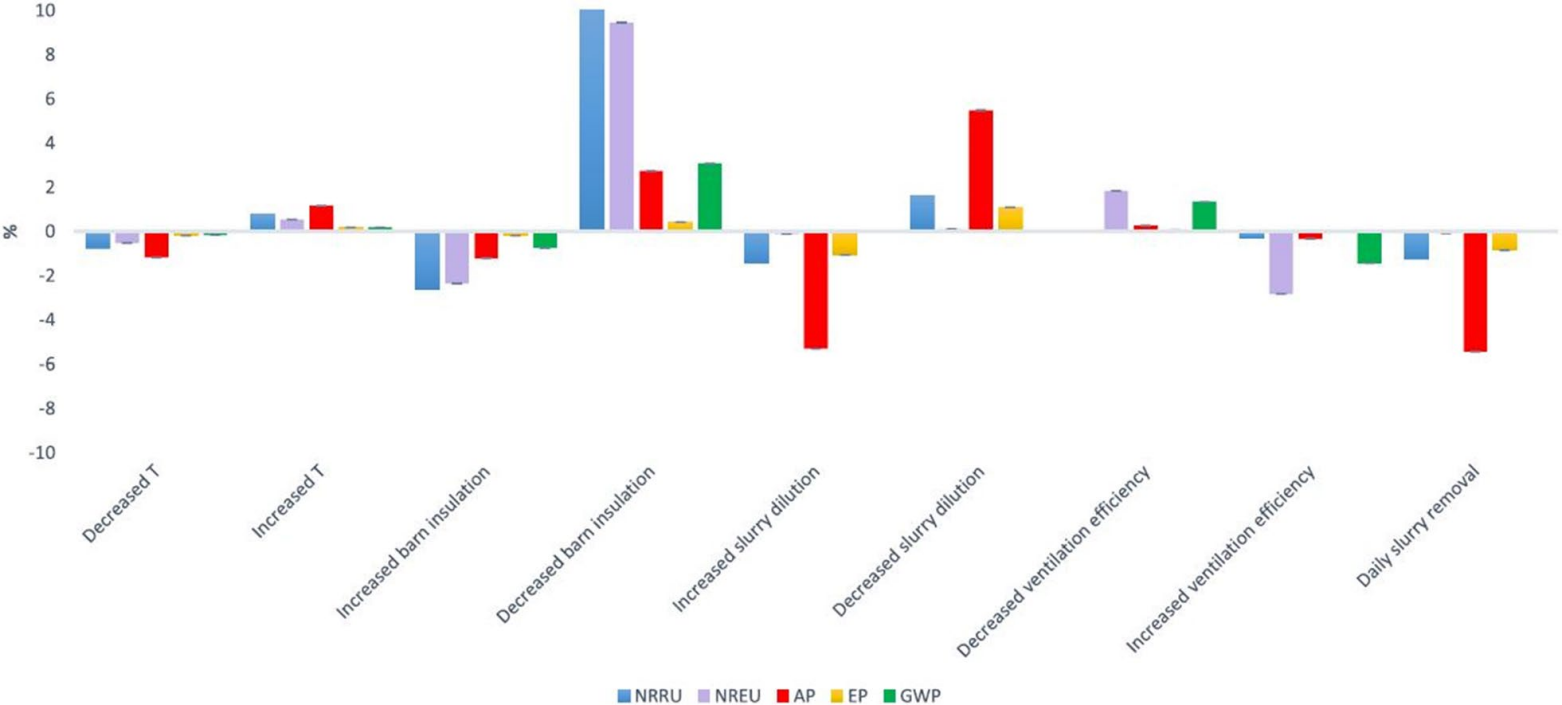
Potential changes in Danish pig systems environmental impact (% change in a specific environmental impact category) by modifications in pig housing infrastructure, management, and technologies (source: Pexas et al. [98]). T = pig housing temperature as regulated by the indoor climate control system and set by the farm manager (i.e., management practice); NRRU = Non-Renewable Resource Use; NREU = Non-Renewable Energy Use; AP = Acidification Potential; EP = Eutrophication Potential; GWP = Global Warming Potential

Effective in-barn climate control is essential for optimal animal growth and performance, particularly the regulation of pig housing temperature and air-flow at pen level in densely stocked industrial pig production systems. For example, Pexas et al. [98] found that increasing in-barn temperature by 10% can lead to approximately 1.5 to 2% higher environmental impact for AP due to increased ammonia emissions, whereas lowering in-barn temperature by 10% has the exact opposite effect (Fig. 6). In addition to effectiveness, the efficiency and environmental sustainability of climate control systems can become increasingly relevant when considering emerging climate change issues (e.g., increasing ambient temperatures). This can be achieved through a combination of management practices, technologies, and infrastructure such as: (i) herd management to improve air circulation at pen levels, (ii) pig showering systems and cooling strategies (e.g., directed ventilation for improved air circulation at pen level) to prevent or combat heat stress events, (iii) smart climate control systems that consider carbon and N emissions alongside temperature and humidity, and (iv) improved barn insulation to minimise heat losses (Fig. 6) [98, 101]. Although improving indoor climate control has been associated with significant reductions in pig system environmental impact, overall energy efficiency, and cost-effectiveness, such solutions are also often hindered by the disrupting factors discussed above.

#### Indoor slurry management and removal of associated emissions

Considering the emission pathways associated with indoors slurry handling, literature suggests that the following practices can significantly improve pig system environmental sustainability: (i) frequent slurry removal from the pig pens (from pen to slurry pit) and slurry pits (from slurry pits to outdoor storage) reduces the potential for ammonia volatilisation when slurry is in contact with air for prolonged periods, (ii) slatted pen flooring to allow slurry flows from pens to pits, thereby reducing ammonia emissions by up to 35%, (iii) bedding material (e.g., straw) that is frequently renewed can also reduce ammonia formation from slurry, (iv) acidification of slurry to reduce ammonia emissions by up to 67% because ammonia volatilisation takes place in alkaline environments (pH greater than 8), (v) air cleaning systems such as wet acid scrubbers to remove up to 80% of ammonia emissions by re-liquidating gaseous pollutants, (vi) slurry dilution to reduce ammoniacal nitrogen concentrations and therefore potential ammonia volatilisation at slurry pits, and (vii) the use of nitrification inhibitors, such as nitrapyrin or 3,4-dimethylpyrazole phosphate, to slow down the conversion of ammonium to nitrate by *Nitrosomonas* bacteria [20, 97, 98, 131]. While as discussed CH_4_ emissions are largely affected by the content of crude fibre and volatile solids in the manure, cooling of slurry through any of the above practices and frequent removal (less storage time) can also help reduce CH_4_ emissions at the pig housing and storage levels, as evidence in literature suggests that methanogenic bacteria become more potent under higher temperatures and the more moist the manure or storing environment is. Further, a neutral pH favours CH_4_ production and therefore, acidification of slurry could help drive emissions lower [103].

#### Outdoor manure management

The processes and resources required for outdoor manure management are responsible for the bulk of emissions associated with the second largest contributor to pig system environmental impacts. As discussed previously, they involve the storage of slurry in large tanks for several months and its application at fields as a fertiliser for crop production. Technological advancements have achieved great environmental impact abatement potential at this production stage, and several multi-fold benefits of their application also arise through indirect circular streams and interactions with the energy and crop production sectors as will be discussed below. Some of the most well-established manure treatment solutions towards system environmental sustainability at this stage involve the separation, and the anaerobic digestion of slurry.

The separation of slurry in a solid and a liquid fraction through various practices and technologies (e.g., screw press, decanter centrifuge) allows for more controlled nutrient redistribution at manure application [122]. Although nutrient separation efficiencies vary depending on the psychochemical properties of manure (e.g., dry matter content) and the separation method used, liquid fractions can contain up to 74% less P compared to the untreated slurry [95, 142]. This makes the strategy particularly relevant for improving efficiency of manure application from pig systems located near phosphorus and nitrogen vulnerable zones [100]. Further improvements can be achieved when combining slurry separation with slurry acidification or additives in pig slurry (e.g., biochar); these improve speed and efficiency of separation through changes in the physicochemical properties of slurry (e.g., lowered slurry viscosity and particle charge), besides contributing to larger reductions in NH_3_ and P at field application [19].

While slurry separation is a popular and well-researched manure treatment method, its implementation at wider scales is hindered several factors such as (i) relatively poor cost-effectiveness, (ii) long-distance transportation requirements for application of phosphorus rich solid fraction to avoid surplus of nutrients in small areas, and (iii) negative effects of climate change on emissions from storage of solid fractions [98–100]. Separating the solid from liquid fraction of slurry and following different storing practices for each one (i.e., slurry tanks vs. covered solid manure) results to reduced CH_4_ emissions, since this way manure (especially the solid fraction) can be stored and treated at lower temperatures and controlling its direct exposure to air more efficiently (i.e., impermeable covers and rapid application in fields) [64].

The other popular manure treatment strategy that helps reduce pig system environmental impact is the anaerobic digestion of slurry or co-digestion alongside a range of feedstocks. The process leads to the production of heat and electricity from biogas, which can be discounted from on-farm energy use or supplied in the national grid. It also returns a nutrient enriched digestate, the chemical properties of which depend on nutrient concentrations of the slurry and nutrient profiles of feedstocks-substrates. The physicochemical properties of the digestate make it a more efficient organic fertiliser than untreated slurry, which when applied at crop production enhances nutrient uptake by the crops while reducing N and P associated emissions [142]. Studies on the integration of anaerobic digestion in EU pig manure management, have found that it can help reduce energy use and GWP associated with pig systems by 40% and 9.24% respectively compared to a baseline pig system where manure is simply stored and applied on the fields untreated [98]. Cherubini et al. [17] investigated the environmental benefits of anaerobic digestion in Brazilian pig systems and found similar results with 11.9% reductions in CO_2_ system emissions. Anaerobic digestion can reduce CH_4_ emissions by ~ 35% compared to untreated slurry, while not much evidence is available to suggest a significant reduction of specific N_2_O and CO_2_ direct emissions; this suggests that observed improvements in system environmental performance when implementing anaerobic digestion are potentially explained by (i) the discounts in energy–fossil fuel use due to biogas production and (ii) discounts in synthetic inputs associated with crop production due to application of the nutrient rich digestate [64, 98]. As discussed previously, the anaerobic digestion technology can be effectively paired with circular feed production alternatives, such as insect farming or the production of cellular protein, to unlock further potential for reduction of pig system environmental impacts.

When exploring the potential implementation of anaerobic digestion to reduce pig system environmental impacts, it is important to consider that its effectiveness and eco-efficiency may vary significantly depending on the mixture of feedstocks used. Lijó et al. [67] for example, found a range of 152 to 619 kg CO_2_ (climate change potential) and 49.9 to 358 m^2^ (arable land use potential) per MWh produced when different feedstock mixtures were used at farm-scale anaerobic digestion plants in Italy. While anaerobic digestion has great potential to be an environmentally sustainable and cost-effective solutions for large pig production systems, pig producers are often deterred from implementing it due to the large investment costs it involves [89]. A system of centralised facilities, like the one that operate in Denmark, could support waste management of pig production systems enhancing environmental sustainability of the sector at broader scales (e.g., at regional or even national levels) [99, 100]. Other bottlenecks to this strategy may be associated with how sensitive the biogas generation potential and digestate efficiency can be against changes in slurry composition (e.g., dry matter levels, presence of bedding material) and ambient climate conditions (e.g., increased temperature greatly increases loss of nutrients at digestate storage) [11, 140].

## Conclusions and the way forward

Literature suggests that the potential environmental implications associated with pig systems are already relatively low, compared to other livestock systems such as beef and dairy cattle [14]. However, it is expected that the popularity and demand for pig meat will significantly increase over the next 25 years (2050) to such an extent that there is an urgent need to identify strategies towards improving the environmental sustainability of current pig production systems.

The aim of this review was to discuss some of the more concerning environmental issues associated with conventional pig systems and a projected intensification of the pig production sector, and present potential solutions that may help reduce such potential environmental impacts along with the bottlenecks needing to be addressed for an effective implementation of solutions at large scales. Some of these solutions already exist and have been implemented in pig systems across the globe. However, the benefits of existing solutions may not be harnessed in full if they are implemented only in smaller scales, in specific system components, and often with a narrow focus of reducing very specific emissions (e.g., methane emissions from enteric fermentation). For example, there has been a very strong focus on feed related interventions to reduce GHG emissions and work towards Carbon Net Zero policies, that came at the expense of impacts arising from N and P excretion (e.g., acidification and eutrophication impacts due to manure application)—a major environmental impact hotspot for pig systems. Currently, there is a lot of discussion about the potential contribution of animal health improvements to the environmental impact of pig systems [74], however, much of it fails to account for potential unintended environmental and economic implications of management practices that are required to ensure animal health and welfare (e.g., climate control system operations,in-barn air filtering systems) and disposal of waste from large scale pig production (e.g., operation of manure management technologies) [99].

The findings of this review suggest that when aiming to maximise environmental impact abatement through interventions at a pig farm level, potential component interactions should be considered. Specific pathways that seem to offer solutions to addressing key environmental issues are the use of existing alternative feed ingredients, particularly to source protein for example from home grown legumes, food waste, and insect meals, and adopting good slurry management practices at pig housing, manure storage and application. The latter could be as simple and cost-effective as removing slurry from slurry pits at a frequent rate e.g., biweekly as opposed to once every month, or could require larger investments but return also larger environmental and economic benefits e.g., anaerobic digestion of slurry for production of biogas and nutrient rich fertiliser.

Future research should adopt a holistic approach to further enhance the quantity and quality of information regarding: (i) the potential sustainability benefits of sustainability solutions at various pig system components, (ii) limits to improvement of pig system components (e.g., genetic selection), (iii) bottlenecks for the stand-alone and joint implementation of potential solutions, and (iv) potential synergistic and antagonistic effects when joint implementation of multiple solutions is considered. Ultimately, this information will facilitate the development of holistic sustainability assessment and decision support tools for pig farm management that account for interactions between the “feed * animal * manure” system components and trade-offs between sustainability priorities (e.g., environmental vs economic performance of pig system; welfare improvements vs environmental impacts). Further, future research should focus on exhaustive, integrated, and prospective LCA studies that investigate the performance of combinations of proposed solutions across different system components, while accounting for geographic and climatic variability to offer solutions towards resilient pig systems [100].

## Data Availability

Not applicable.

## Abbreviations

AP: Acidification potential
EP: Eutrophication Potential
GHG: Greenhouse Gases
GWP: Global Warming Potential
LCA: Life Cycle Assessment
LU: Land Use
NREU: Non-renewable Energy Use
WRU: Water Resource Use
